# Advance Directives: Knowledge of the Topic Among Psychiatrists

**DOI:** 10.3389/fpubh.2022.822577

**Published:** 2022-02-23

**Authors:** Vania Novelli Domingues, Luísa Castro, Monica Domingues Monteiro, José Antonio Cordero da Silva, Francisca Rego, Guilhermina Rego

**Affiliations:** ^1^Faculty of Medicine, University of Porto, Porto, Portugal; ^2^Faculty of Engineering, State University of Rio de Janeiro, Rio de Janeiro, Brazil

**Keywords:** advance directive, pandemics, psychiatry, terminal disease, therapeutic obstinacy, patient autonomy

## Abstract

**Objective:**

Advance directives are becoming increasingly important as health technologies evolve. We sought to assess psychiatrists' knowledge of advance directives, as this knowledge is fundamental to the implementation and drafting of these personal documents.

**Methods:**

A previously published questionnaire that evaluated the knowledge of medical professors was used. The sample, composed of psychiatrists from Rio de Janeiro, Brazil, originated from a publicly available list. During the search process, the COVID-19 pandemic affected Brazil and the rest of the world, which influenced our methodology and results.

**Results:**

A total of 40 psychiatrists participated in the study. The results obtained, although not significant, suggested that psychiatrists with an increased time of practice had more knowledge of advance directives. Nevertheless, less than half of the participants had knowledge about this topic.

**Conclusion:**

The number of psychiatrists surveyed indicates the need for further studies on the subject. The influence of the COVID-19 pandemic on this study led to findings such as a change in sensitivity when addressing the topic and greater difficulty in contacting professionals.

## Introduction

In ancient Rome, citizens could refuse medical treatment and even choose death, provided that within the culture of the time they were considered rational and able to assess the consequences of their act (1). In the current context, where death has been displaced to the hospital environment and technology can lead to disproportionate treatments, it is necessary to reflect on basic concepts (2).

Disproportionate medical interventions that do not seek patient recovery but rather seek to prolong life beyond what is reasonable and without quality of life for the patient constitute a form of malpractice known as therapeutic obstinacy (3). It is more important now than ever before to honor patients' wishes when facing the possibility of a long-term disease that demands hospital admissions and various medical interventions, which is exactly the purpose of advance directives: they are instruments that enable patients, even when they reach the terminal phase, to die with dignity (4).

In the United States, the Patient Self-Determination Act, effective December 1, 1991, stipulates three forms of advance directives, namely: the living will, which defines the treatments the individual agrees or does not agree to undergo; the durable power of attorney for health care, which defines the chosen representative in health care matters who will make decisions on the patient's behalf when they can no longer do so themselves; and the advance care medical directive, which is more comprehensive, integrating the first two forms (4).

Chapter II, Article 9 of the Convention for the Protection of Human Rights and Dignity of the Human Being with regard to the Application of Biology and Medicine clearly states: “The previously expressed wishes relating to a medical intervention by a patient who is not, at the time of the intervention, in a state to express his or her wishes shall be taken into account” (5). The Brazilian Federal Council of Medicine (CFM) has taken a stance on advance directives through resolution CFM 1995/2012, providing some support for doctors who are in the process of following or suspending a certain therapeutic path in accordance with the previously expressed wishes of the patient. Some legal uncertainty still remains, however, because although advance directives have a specific resolution, there is no legal provision for them in Brazilian legislation (6).

The Civil Code of Brazil values the autonomy of the citizen and specifically provides for wills, but they are restricted to material goods (property); living wills are not provided for and are non-existent (7). In addition to legal uncertainty, which is an important obstacle to the application of advance directives, there are some barriers on the part of physicians themselves, namely lack of training in dealing with the end of life. It has been suggested that the medical curricula should promote the development of skills to deal with families and patients in a broader sense, such as developing an empathic relationship and involving and informing the family and patient in the process, due to complex feelings of guilt or even beliefs that often lead them to postpone the final outcome indefinitely (8). Also, not knowing the diagnosis and prognosis makes it difficult to make a decision that may be fundamental for the patient (8).

Although the limitation of life support, which is based on not applying or even on suspending therapies that prolong the life of terminal patients with no chance of recovery, is technically considered a medical issue, family involvement in the process is required (9). To that end, the CFM issued a resolution to support doctors in this type of delicate situation, highlighting their obligation to explain the procedure to the patient, if possible, and to the family member or legal representative, in addition to recording it in medical records (10).

It is important to remember that advance directives are highly dependent on the quality of information provided to the patient; it is essential an attending physician participates in order to assist the patient and clarify any doubts about the resources that may be used. In fact, without this, the advance directive will not represent the patient's convictions, as the patient will not have understood their disease, its probable evolution, and the possible treatments (11).

It is possible to plan treatments within advance directives, although some of these may be considered “outdated therapies” at the time the advance directive is applied, at which point they will be “considered null, with a view to preventing the living will from putting the interests of the patient at risk” (11). Efforts were made to create a model advance directive based on examples from other countries that would be representative of the Brazilian reality. The result was a document that values subjectivity in order to assist the patient and the physician and thus bring greater comfort. It is more a guide than a model (12).

It is very important to emphasize that patients' ability to understand and make decisions for themselves is fundamental to drawing up an advance directive that may actually achieve its proposed objective. Furthermore, the diagnosis of a disease that, in its presumed evolution, tends to compromise a patient's reasoned decision-making capacity should be accompanied by actions that stimulate the elaboration of an advance directive while the patient is still able (2).

It is necessary to remember that facing the condition of being near to death raises defense mechanisms, as described by Elisabeth Kübler-Ross, who outlines five such mechanisms in addition to the feeling of hope, which is the feeling that tends to persist throughout the process and is an important ally in the quality of life of terminally ill patients. “Nor does he give up hope that some cure may be discovered, that a new medicine may be produced in time to relieve him of his sufferings” (13).

## Objective

The present study aims to evaluate the knowledge of advance directives among psychiatrists from the municipality of Rio de Janeiro, Brazil, in addition to using this opportunity of contact to raise awareness of the subject among those who do not know about it and introduce them to Resolution 1995/2012 (6).

### Methodology

This is an observational, cross-sectional, and quantitative study based on a questionnaire administered to psychiatrists by a principal investigator and a co-investigator between December 13, 2019, and December 10, 2020.

### Participants

Psychiatrists from the municipality of Rio de Janeiro who had authorized their names and, in most cases, addresses and telephone numbers to be disclosed to the general public on the Brazilian Psychiatry Association (ABP) website, from which the list was compiled on June 28, 2019, were considered to be included. The population of respondents was obtained from the list available to the public through the Brazilian Psychiatric Association.

At the time, there were 265 registered psychiatrists on the list, of which 213 had agreed to make their names, addresses, and telephone numbers public. Excluding the principal investigator, who appeared on that list, 212 psychiatrists comprised the group to be researched.

### Procedures

Potential respondents were contacted by telephone to schedule a face-to-face meeting for the application of the questionnaire at a location considered appropriate by the psychiatrist. The contacts were collected from a publicly available list from the Brazilian Psychiatric Association. Psychiatrists who did not respond or did not agree to schedule a meeting were considered excluded.

In the cases where the meetings were scheduled, the researchers followed the script they had practiced. At the end, the respondents were asked if they had any doubts and, if so, were given due clarification.

The principal investigator and the co-investigator presented Resolution 1995/2012 of the CFM, revealing that the resolution would be the main research subject. The free and informed consent form was presented shortly afterward. Before the meeting and after reading the free and informed consent form, the latter was signed in duplicate, with one copy remaining with one of the researchers and the other with the respondent. The questionnaire was then administered, which, because it was anonymous, was completed by the researchers to avoid including identifying information. The researchers kept the questionnaires for archiving. Care was taken to maintain a uniform standard regarding the terms used.

At the beginning of March 2020, the methodology for administering the questionnaires became essentially infeasible due to the COVID-19 pandemic that overtook the country.

Since the objectives of the research were unchanged, the adaptation of the methodology to the new reality was approved on July 17, 2020, by the Research Ethics Committee (REC), with the following changes: when contact was made by telephone, resolution CFM 1995/2012 was sent by electronic means, as was the free and informed consent form. When consent was obtained from respondents, they were asked if they had any doubts, and if so, these were resolved. After that, the electronically signed free and informed consent form was received, then printed and filed. The meeting was scheduled remotely, and one of the investigators filled in the questionnaire, which was archived.

Limitations due to the COVID-19 pandemic: data collection was suspended on March 12, 2020, because of the pandemic, and only one in-person meeting was held, on July 15, 2020, with adherence to all due precautions of keeping about two meters' distance and using masks, since the REC had not yet given permission for remote meetings. The research was suspended for over 4 months due to the risk of contamination by COVID-19. On July 17, 2020, the REC authorized continuing the research remotely, given that the research objectives had not changed. This change in methodology and the pandemic situation had various influences, which will be outlined in the results.

### Instruments

The questionnaires were divided into five sociodemographic questions and two open-ended questions, which were included for a later study. They were: informed consent and participant information; sociodemographic questionnaire (gender, marital status, religion, education, and training in medicine and psychiatry); questionnaire on advance directives: 10 questions originating from a questionnaire used in a previously published study with Brazilian medical school professors (14), namely on the knowledge of advance directives and the laws regulating them; discussing the subject with relatives or other persons the psychiatrist has contact with; the influence of religion on these issues; conduct that the psychiatrists would personally adopt with their families in situations of irreversible disease and end of life; respect for decisions made by the family and health professionals.

### Statistical Analysis

The data collected were managed using Microsoft Excel 2016, and statistical analysis was performed using SPSS® Statistics (version 26.0; SPSS Inc., Chicago, IL, USA). Categorical variables were described by absolute and relative frequencies, *n* (%). Quantitative variables were described using medians and interquartile intervals [1stQ; 3stQ]. The Mann-Whitney *U*-test or Kruskal-Wallis test was used to compare quantitative variables between two or three groups, respectively. *P*-values were considered significant if <0.05.

## Results

Forty psychiatrists were included in the study; 52.5% were male and 42.5% were married or living with a partner. Forty percent were religious, namely Catholic, followed by 32.5% who claimed to have no religion. The median time since medical graduation was 41 years, and the median time since psychiatry specialization was 34 years (Table 1).

**Table 1:** Sociodemographic characteristics of the 40 participants.

**Variable**	**Description**
**Gender**	***n***(%)
Male	21 (52.5)
Female	19 (47.5)
**Marital status**	***n***(%)
Single	8 (20)
Married/living with partner	17 (42.5)
Divorced/separated	15 (37.5)
**Religion**	***n***(%)
Catholic	16 (40)
Evangelical	3 (7.5)
Spiritualist	2 (5)
None	13 (32.5)
Other	6 (15)
Time since graduation (years), med [IQI]	41 [33.25; 44]
Time since specialization (years)*, med [IQI]	34 [25; 41]

MED, median; [IQI], range from 1st to 3rd quartiles; *five respondents did not remember when they became specialized in psychiatry.

The distribution of the answers of the 40 psychiatrists participating in the advance directives questionnaire is shown in Table 2.

**Table 2:** Responses of the 40 participants to the Advance Directives Questionnaire.

**Question**		***n* (%)**
Do you know what an advance directive is?	Yes	18 (45)
	No	15 (37.5)
	Some idea	7 (17.5)
Do you know if there is any law in Brazil that regulates advance directives?	Yes	15 (37.5)
	No	25 (62.5)
Has a family member ever talked to you or someone else about advance directives?	Yes	22 (55)
	No	18 (45)
	I do not know	0 (0)
Do you think that religion can interfere with these decisions?	Yes	37 (92.5)
	No	2 (5)
	I do not know	1 (2.5)
Do you think of drafting an advance directive?	Yes	24 (60)
	No	6 (15)
	I do not know	10 (25)
If your family member asked to die at home, would you accept it, or would you prefer the hospital?	I would not accept it	9 (22.5)
	I would accept it	21 (52.5)
	I do not know	10 (25)
If you were in terminal phase, would you prefer to be treated at home or in hospital?	Hospital	13 (32.5)
	Home	17 (42.5)
	I do not know	10 (25)
Would you stay in the Intensive Care Unit at the end of your life?	No	20 (50)
	Yes	13 (32.5)
	I do not know	7 (17.5)
Do you believe your family members would respect your decision?	No	0 (0)
	Yes	32 (80)
	I do not know	8 (20)
Do you believe that medical teams would respect your decision?	No	2 (5)
	Yes	25 (62.5)
	I do not know	13 (32.5)

Among the respondents, 45% knew what advance directives are, but 62.5% did not know the law that regulates them. Also, 55% had already talked about advance directives with family or other people.

Most participants, namely 92.5%, thought that religion interferes with decision-making regarding advance directives. Sixty percent of respondents reported thinking about drafting their own advance directives. As for their position on a family member's request to die at home, 52.5% declared they would accept it; however, when it comes to where the respondent themselves would prefer to receive care in a terminal phase, 42.5% said they would prefer to be cared for at home, 32.5% preferred a hospital, and 25% did not have a preference. As for whether or not the respondent would accept staying in an Intensive Care Unit at the end of their life, 50% said “No,” 32.5% answered “Yes,” and 17.5% did not know. As for family members not respecting their (the respondent's) decision, it is interesting that 0% of respondents considered this possible. As for medical teams respecting their (the respondent's) decision, 62.5% believed their will would be respected.

Table 3 shows an increased knowledge of advance directives among participants in accordance with the duration of training in medicine, but without statistical significance. This means that participants with knowledge of advance directives had completed their training in medicine longer ago than participants without knowledge of advance directives.

**Table 3:** Comparison of the time since graduation (years) of participants according to advance directives questionnaire responses.

	**Do you know what an advance directive is?**			
	Yes	No	Some idea	Kruskal Wallis' *P*-value
Time since graduation (years), med [IQI]	42.5 [35; 46.5]	36 [30: 41]	41 [34; 41]	0.087
	**Do you know if there is any law in Brazil that regulates advance directives?**			
	Yes	No	Mann-Whitney's *P*-value
Time since graduation (years), med [IQI]	41 [35; 45]	41 [33; 43.5]	0.489
	**Do you think of drafting an advance directive?**			
	Yes	No	I do not know	Kruskal Wallis' *P*-value
Time since graduation (years), med [IQI]	40.5 [35; 43.75]	32.5 [21.25; 51.75]	41 [33; 45]	0.611

*MED, median; [IQI], range from 1st to 3rd quartiles*.

### Final Status

**Figure 1:**
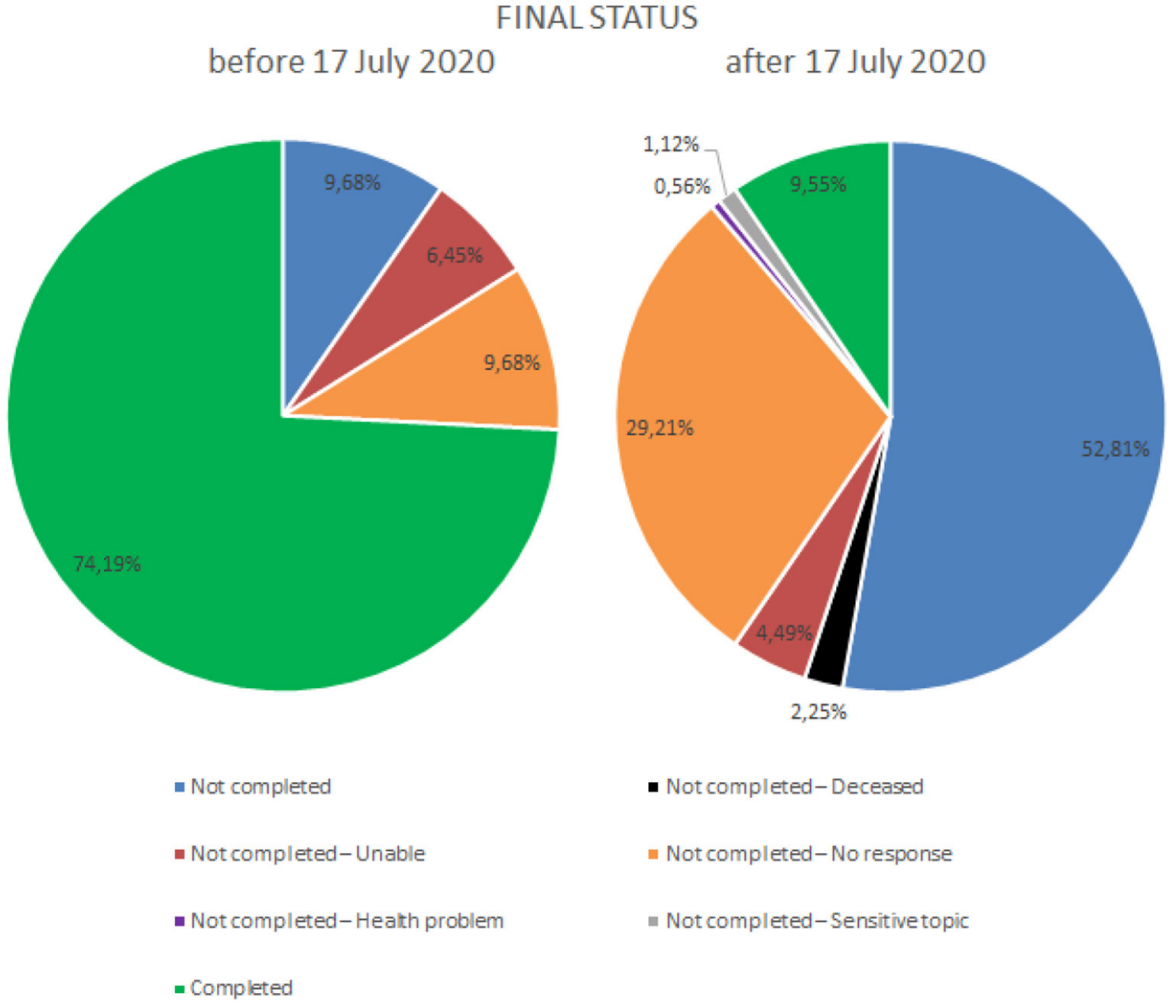
Comparison chart of completion status before and after July 17, 2020.

In Figure 1 describing the final status, we sought to explore the influences that occurred in the data collection process, comparing the period before July 17, 2020, and the period after July 17, 2020. There were three main reasons for not completing the questionnaire that only appeared after July 17, 2020, being absent in the previous period: death, sensitive topic, and health problems.

## Discussion

The International Convention on the Rights of Persons with Disabilities (UN) (15) brought a new way of dealing with civil capacity and with case study analysis, especially in the case of forensic psychiatrists, as well as influencing the Statute of the Person with Disabilities that came into force on 2016 in Brazil. According to this new statute, the patient with a mental disability, which includes all mental illnesses, such as schizophrenia, bipolar disorder, depression or dementia, is considered disabled and is guaranteed full civil capacity. Meaning that mental disability can no longer compromise a person's full civil capacity. Thus, promoting a condition of equality between disabled and non-disabled regarding acts of civil life, namely conducting an advanced care directive, provided that there is no judicial decision of determination of incompetence (16).

The aim of this study was to assess the knowledge about advance directives of psychiatrists in the municipality of Rio de Janeiro, Brazil. As a secondary objective, this study aimed to disseminate information on advance directives to those who did not know about them.

Advance directives are an important instrument to empower and promote patient autonomy and to actively involve patients in the healthcare decision-making process. Besides providing important clinical information, they improve the therapeutic relationship, enable communication about past treatment experiences, and encourage discussion of preferences and choices for future treatments (17–19).

Furthermore, they help the clinician to better manage and care for the patient, according to the patient's will and wishes, therefore reducing the use of excessive/involuntary treatments and the length of hospitalization (20, 21).

The finding that we can highlight is that the greatest knowledge about advance directives was found among respondents with more time in professional activity. This finding is different from the previous study among medical teachers and students and patients' caregivers (14), in which those who did not know about advance directives were older. But when it came to professional activity, the students had more knowledge than the teachers, and the teachers had more than the patients' caregivers. It was discussed that the fact that students had more knowledge about advance directives than teachers was associated with changes in the curriculum and the area of professional activity (14). On the other hand, another study conducted with psychiatrists, psychologists, and mental health social workers found that age and endorsing positive perceptions of advance directives were associated with fewer perceived barriers to implementing those directives (22).

In this study, fewer than half of those surveyed had knowledge about advance directives, and most were unaware of the law that regulates them. Given that the sample is skewed toward psychiatrists with more years of practice and experience and that greater knowledge about the topic is found among participants with more years of professional practice, this can explain the low rates of knowledge on advanced directives found in this study. Furthermore, most respondents believed that religion interferes with decision-making regarding advance directives. An important factor that compounds the difficulty of developing advance directives is the great lack of knowledge of this subject among clinicians (14). If the doctors themselves are uninformed on the subject, they will not be able to assist patients in drafting an advance directive, inform patients of this right, or fulfill the desires outlined in the document. Moreover, the lack of laws regulating the subject brings, even to those who are aware, legal uncertainty (14).

In order for psychiatrists to engage in advance directives, it is essential to increase education on the topic and on the key aspects of advance directives legislation, as well as to facilitate access and support for implementing advance directives (23). Advance directives are comprehensive tools that allow psychiatrists to collect patients' beliefs and cultural values and translate them into their wills and treatment preferences, as well as reinforcing patient-family relationships through collaborative and surrogate decision-making in case of crisis (24).

An unexpected finding of this study was the change in sensitivity when approaching the advance directives theme, linked to finitude and greater difficulty in contacting physicians, when we resumed the research after July 2020, when the pandemic COVID-19 was a reality.

The argument that advance directives would be a sensitive subject that respondents preferred not to address only appeared in the period following the pandemic. Could there be a rising awareness regarding the end of life, which is ultimately what advance directives address? Following this line of reflection, it has been cited that deaths caused by COVID-19 affect mental health more than other natural-cause deaths. There would be a tendency for end-of-life matters to become more present in people's lives (25), perhaps making them more sensitive to the subject. It is a reflection to be considered.

There is also reference to the negative effects that experiences of suffering have on people with or without mental disorders, and while this effect is more intense in patients who had mental disorders prior to the COVID-19 pandemic, it is not restricted to them. In addition to losses of friends and relatives, financial problems and social isolation are mentioned, as are work activities (25).

People have seen large losses in their lives in various areas of social relations. The Brazilian Association of Psychiatry (ABP) undertook several actions during this period, forming partnerships with federal agencies and autonomously seeking to support and guide the population and health professionals.

Medical training is another critical issue to point out, as it needs reformulation. Many factors are associated with the difficulties that were encountered, such as academic training toward curative practice, which is not always possible, and viewing death as a professional failure (26).

Continuing with the analysis of the relationship of medical training and communication difficulties between doctors, patients, and family members, we return to the studies developed by Elisabeth Kübler-Ross, which brought great contributions to questions regarding the end of life and the feelings that permeate human relations in the face of death. She defined five stages of facing death: denial and isolation, anger, bargaining, depression, and acceptance. These could be felt by patients and even family members, but mainly, and in relation to the theme that we are specifically addressing, she aptly spoke about the feelings and reactions of healthcare teams. She identified the physician's difficulty in dealing with death itself as an important reason for patient abandonment and also the reason for many of the difficulties observed in treating terminal patients; within the healthcare team, those who resisted the subject the most were the physicians (13).

Another important contribution was the perception of the importance of being truthful when communicating with patients. She suggested sharing information about the disease with the patient, in a way, by discussing therapeutic possibilities and strategies. She also gave value to the possibility of creating hope, in the sense of the disease being a shared struggle, a battle to be fought by the doctor and the patient, writing that the patient “will not be abandoned,” which she expressed in this way: “the important thing is to communicate to the patient that not all is lost; that this is a battle that has to be fought together—patient, family and doctor—regardless of the final outcome” (13).

All of these aspects must be considered, because according to the Brazilian Medical Ethics Code, Chapter V, Article 34, it is up to the physician to “inform the patient of the diagnosis, prognosis, risks and objectives of the treatment, except when direct communication may cause damage to the patient, in which case the information must be communicated to their legal representative” (27). Therefore, the physician must prepare to perform this duty in the technical, ethical, and even emotional sense (13).

To that end, a procedure was developed with the objective of helping and preparing the professional for talking with patients when the outcome isn't good: The “SPIKES: A six-step protocol for delivering bad news.” It was designed for use with cancer patients, but it may be important on many occasions, as there are other diseases that may also have presumably unfavorable outcomes. This preparation is considered important for addressing the topic of the end of life (28).

Due to the small number of questionnaires completed, it was difficult to compare the knowledge of psychiatrists with that of practitioners of other medical specialties available in the literature. Increasing and diversifying respondents by Brazilian regions could allow the extrapolation of the results and the exploration of possible biases inherent to the method.

Even though the obtained sample of respondents was relatively low, with all due reservations, it is possible to highlight some influences that the change of approach (in-person to online contact) had on the respondents, as well as some effects of the COVID-19 pandemic. Although this sample may not be representative of the population of psychiatrists in Rio de Janeiro, the lack of studies in this field highlights the relevance of this research. There is also a possible selection bias, given that the psychiatrists who agreed to participate in this study might already have more knowledge or interest in the topic. There may also be a self-reporting bias that overestimated the rate of psychiatrists having knowledge about advanced directives; nevertheless, participation in this study provided an informed clarification of the topic.

In-person contact became contraindicated, and the research was paralyzed for approximately 4 months. Therefore, respondents were contacted remotely upon receiving due authorization by the REC. Having the respondent print and sign the free and informed consent form for archiving was a significant complicating factor, as during the process of being contacted, becoming familiarized with resolution 1995/2012, reading and printing the form to sign it remotely, some respondents gave up, with a difference of almost 20% more dropouts compared to the previous period. Having all the material printed and ready to be signed and filed seems to have been an important facilitator that was lost.

So, more research is required on the knowledge of advance directives, perceptions about and barriers to its implementation by medical psychiatrists so that we can make an assessment that is closer to the actual state of psychiatry and propose strategies to facilitate its adoption in clinical practice.

In conclusion, psychiatrists can be consulted by those complaining of memory changes, and, in cases of a diagnosis of dementia symptoms, the patients can, at least initially, be encouraged to draft advance directives so long as their cognition and awareness allow for it. This is an opportunity that should not be lost (2).

This characteristic of growing knowledge about advance directives in connection with the length of professional practice could also be related to the practice of conducting cognitive evaluations of patients in the initial stages of dementia and “mental health” evaluations, which are common psychiatric practices in Brazil (29), to ensure the validity of documents in general.

The consequences of the COVID-19 pandemic are still being monitored, and their dimensions are still unpredictable. The pandemic has interfered in the research process, both by making physical contact difficult and by making discussion of the topic difficult. In reality, the end of life is a topic that has become more present than ever in people's lives, and avoiding discussing advance directives only makes us more vulnerable. We delegate to others decisions and choices that should be our own, inherent in our autonomy.

In fact, talking about dying with dignity brings us closer not to death, but rather to our choices for preserving the values we consider important. Medical training needs to address end-of-life issues, just as it addresses measures to prevent and cure disease. It also needs to deal with palliative care for patients with terminal diseases.

Just as we are born, 1 day we will die; to deny this fact only weakens us in situations that will foreseeably come at some point in life. The doctor is a fundamental part of raising awareness about and assisting in drafting advance directives.

## Data Availability Statement

The original contributions presented in the study are included in the article/supplementary material, further inquiries can be directed to the corresponding author/s.

## Ethics Statement

The studies involving human participants were reviewed and approved by Faculdades Integradas Aparício de Carvalho-FIMCA. The patients/participants provided their written informed consent to participate in this study.

## Author Contributions

VD and GR contributed to the conception and design of the study. VD and MM performed data collection and LC the statistical analysis. VD, LC, JACS, and FR contributed to the analysis and interpretation of data and bibliographic research. FR and GR did an extensive revision of the manuscript. All authors contributed to the article and approved the submitted version.

## Conflict of Interest

The authors declare that the research was conducted in the absence of any commercial or financial relationships that could be construed as a potential conflict of interest.

## Publisher's Note

All claims expressed in this article are solely those of the authors and do not necessarily represent those of their affiliated organizations, or those of the publisher, the editors and the reviewers. Any product that may be evaluated in this article, or claim that may be made by its manufacturer, is not guaranteed or endorsed by the publisher.
